# Intramyometrial pregnancy after cryopreserved embryo transfer: a case report

**DOI:** 10.1186/s12884-020-2784-7

**Published:** 2020-02-10

**Authors:** Yuan Liu, Yu Wu

**Affiliations:** Reproductive Medicine Center, Department of Obstetrics and Gynecology, Shanghai General Hospital, Shanghai Jiaotong University School of Meidicine, Shanghai, China

**Keywords:** Intramyometrial pregnancy, Laparoscopy, IVF-ET

## Abstract

**Background:**

Intramyometrial pregnancy is a rare subtype of ectopic pregnancy. The cases following IVF-ET were few reported in recent years. The etiological factors include previous uterine trauma like myomectomy, salpingectomy, dilatation and curettage, assisted reproductive technologies and adenomyosis. Early diagnosis is difficult to make due to its various manifestation. The medical treatment includes conservative management with surgical excision, aortic balloon occlusion, uterine artery embolization, MTX etc. Sometimes hysterectomy was performed due to delayed diagnosis.

**Case presentation:**

In this article, we presented a case of a 28 years old woman who had cryopreserved embryo transfer with a history of right side salpingectomy. We suspected it a right adnexa ectopic pregnancy at the first place, especially the right fallopian interstitial or right uterus cornu due to ultrasonography and medical history. The product of conception was discovered embedded in the myometrium and protruding out from the right side of the posterior uterine wall, with seemingly no connection with uterine cavity nor fallopian tubes. The diagnosis of intramural pregnancy was made intraoperatively and validated after pathological report. The interventions were made early enough that exploratory laparoscopy, hysteroscopy and conservative surgical excision were successfully performed at 7 weeks’ gestation preserving the fertility.

**Conclusions:**

It is important for clinicians to be aware of risk factors of intramural pregnancy and maintain an index of suspicion in ART treatment. Ultrasound and laparoscopy are essential managements for early diagnose which make conservative treatment possible and prevent life-threatening consequences.

## Background

Assisted reproductive technology (ART) increases the risk of ectopic pregnancy. The rate of ectopic pregnancy is about 1–2% in spontaneous pregnancy and as high as 1.0–5.4% among those with ART [1]. The majority of ectopic pregnancy occurs within fallopian tube, including fallopian tube interstitial segment, ampulla, infundibulum [2]. There are also non-tubal pregnancy, namely corneal, ovarian, cesarean scar, cervical, intramyometrial and abdominal [3].

Intramyometrial pregnancy is among the rarest subtype of ectopic pregnancy. It was firstly described by Doederlien in 1913 [4]. Intramyometrial pregnancy represents an unusual form of implantation in which the gestational sac is located within the myometrium without any connection to the endometrial cavity, fallopian tubes or round ligament. The majority of intramyometrial pregnancy happens to patients with a history of uterine trauma like myomectomy, salpingectomy, dilatation and curettage, assisted reproductive technologies and adenomyosis [5]. Early diagnosis of intramural pregnancy is difficult to make and most diagnosis is made intraoperatively [6]. Early management with unruptured intramural pregnancy would give these patients the chance to preserve fertility. In this case report, we described an intramyometrial pregnancy following a cryopreserved embryo transfer (CET) with a history of right-side ipsilateral salpingectomy. Institutional review board and ethics committee of Shanghai general hospital approval was obtained. The patient has given her consent for publication of this report.

## Case presentation

**Fig. 1:**
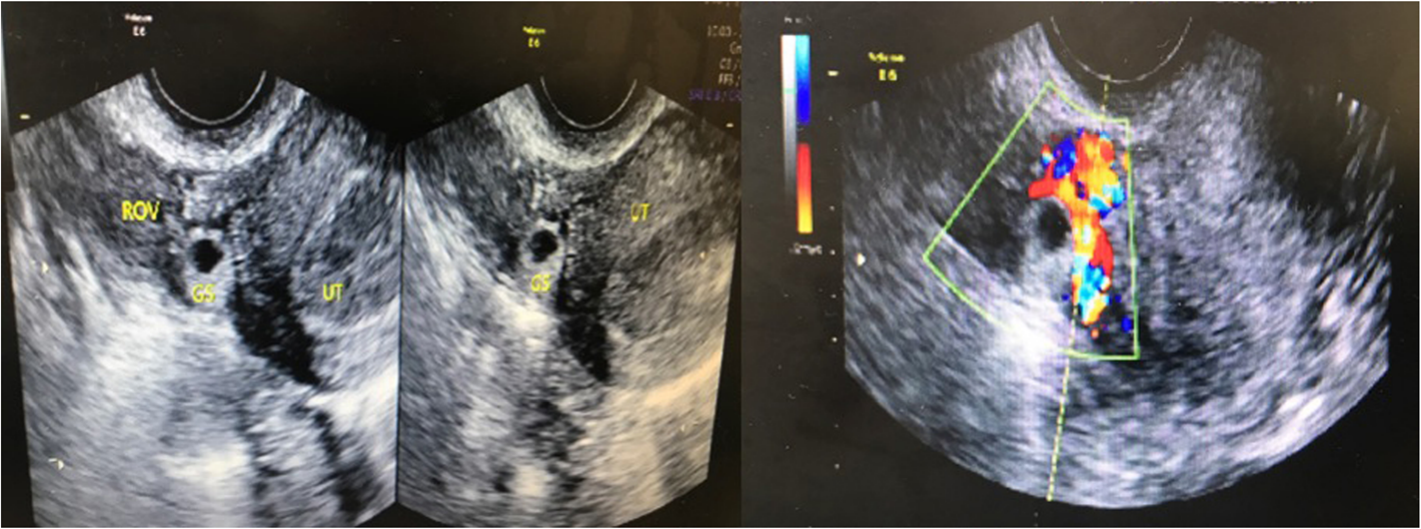
Transvaginal ultrasonography showed the gestation located between uterus and right ovary. Doppler flow parameters showed elevated peritrophoblastic blood flow

**Fig. 2:**
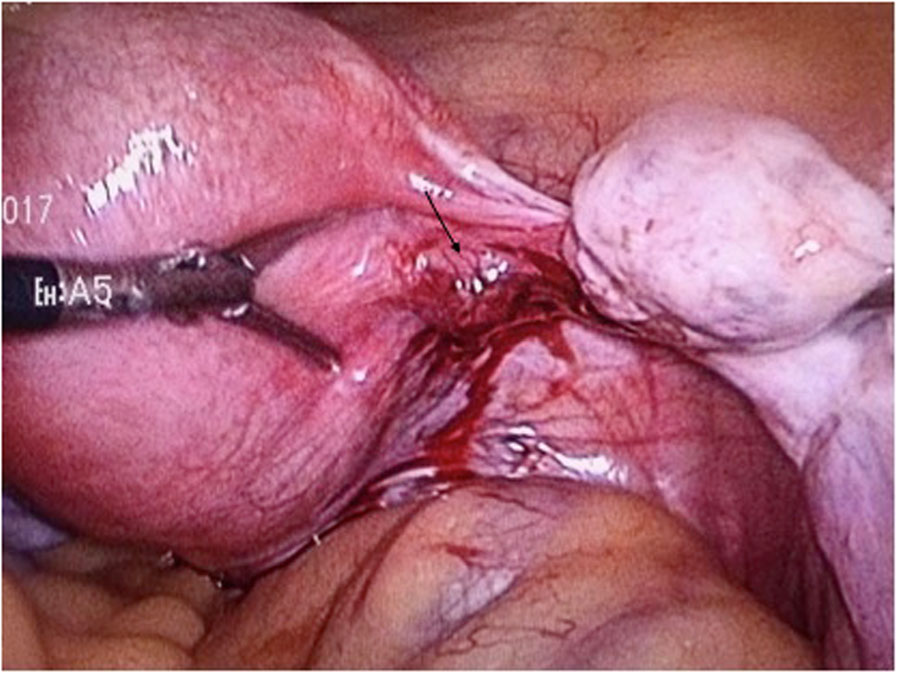
Laparoscopic intra-operative view showed the intramural pregnancy protruding out from the right posterior uterine wall, under the right ovarian ligament. The black arrow pointed the lesion

We presented a case that a 28 year-old woman, gravida 2 para 1, had a history of 2.5 years secondary infertility. Her first pregnancy was full-term normal delivery 6 years ago. The second was spontaneous right fallopian ectopic pregnancy with right-side salpingectomy treatment 3 years ago. Then the patient underwent IVF treatment and had 2 implantation failures in the first cycle of IVF. During her second IVF cycle, she was given a GnRH antagonist regimen for controlled ovarian stimulation. The protocol for the preparation for cryopreserved embryo transfer was hormone replacement treatment (HRT). Afterwards, 2 good quality embryos were transferred into the patient’s uterus under ultrasound guidance. The procedure of embryo transfer was uneventful with soft-tipped catheter, without touching the uterine wall. The serum β-hCG was 42.49 mIU/mL at the 13 day after transfer. The patient presented with no obvious symptoms, just slight protracted dripping. At 7 weeks’ gestation, serum β-hCG rose to 2174.04 mIU/mL but the transvaginal ultrasonography scan revealed an empty endometrial cavity with no sign of fetal pole or yolk sac. However, a suspicious ill-defined hypoechogenic structure with 14*13 mm in size, without sigh of gestation sac nor fetal pulsation, was observed from the ultrasonography. It was seemingly between the right ovary and the uterus contour (Fig. 1). Since the patient described the history of right fallopian pregnancy and right salpingectomy treatment, we suspected a right adnexa ectopic pregnancy, especially the right interstitial fallopian or right uterus corneal pregnancy. Decision was made to proceed with exploratory laparoscopy and hysteroscopy. Under hysteroscopy, no sign of gestation sac was found. Both ostiums from fallopian tubes were seen. There was also slight intrauterine adhesion in the fundus and some decidua tissue hyperplasia. Under laparoscopy, the uterus was enlarged as 7 weeks’ gestation size. The product of conception was discovered embedded in the myometrium and protruding out from the right side of the posterior uterine wall, under the right ovarian ligament, with seemingly no connection with uterine cavity nor fallopian tubes (Fig. 2). The serosa and myometrium above the conception was compressed thin, but not ruptured. The right fallopian tube was missed. The left fallopian through its entire length and both ovaries appeared grossly normal. There was no active bleeding in pelvic cavity. We first injected 5 ml arginine vasopressin with the concentration of 0.6 U/ml into the myometrium. An incision was made over the buldging myometrium from the posterior uterine wall, dark red tissue suggestive of the conception was showed and carefully explored, confirming the conception was not connected to the endometrium cavity. Since the patient described the history of right salpingectomy treatment, we couldn’t confirm if the conception was connected to the right fallopian tube. The diagnosis of intramural pregnancy was suspected during the operation. Then we excised the lesion followed by repairing the defect with careful electrocoagulation. The estimated blood loss during the operation was around 100 ml. The β-hCG level dropped to 4.36 mIU/ml 14 days after the surgery. The pathological report confirmed the presence of chorionic villi in the uterine myometrium, validating the diagnosis of an intramyometrial pregnancy. There was no adenomyosis found from the tissue resected.

**Fig. 3:**
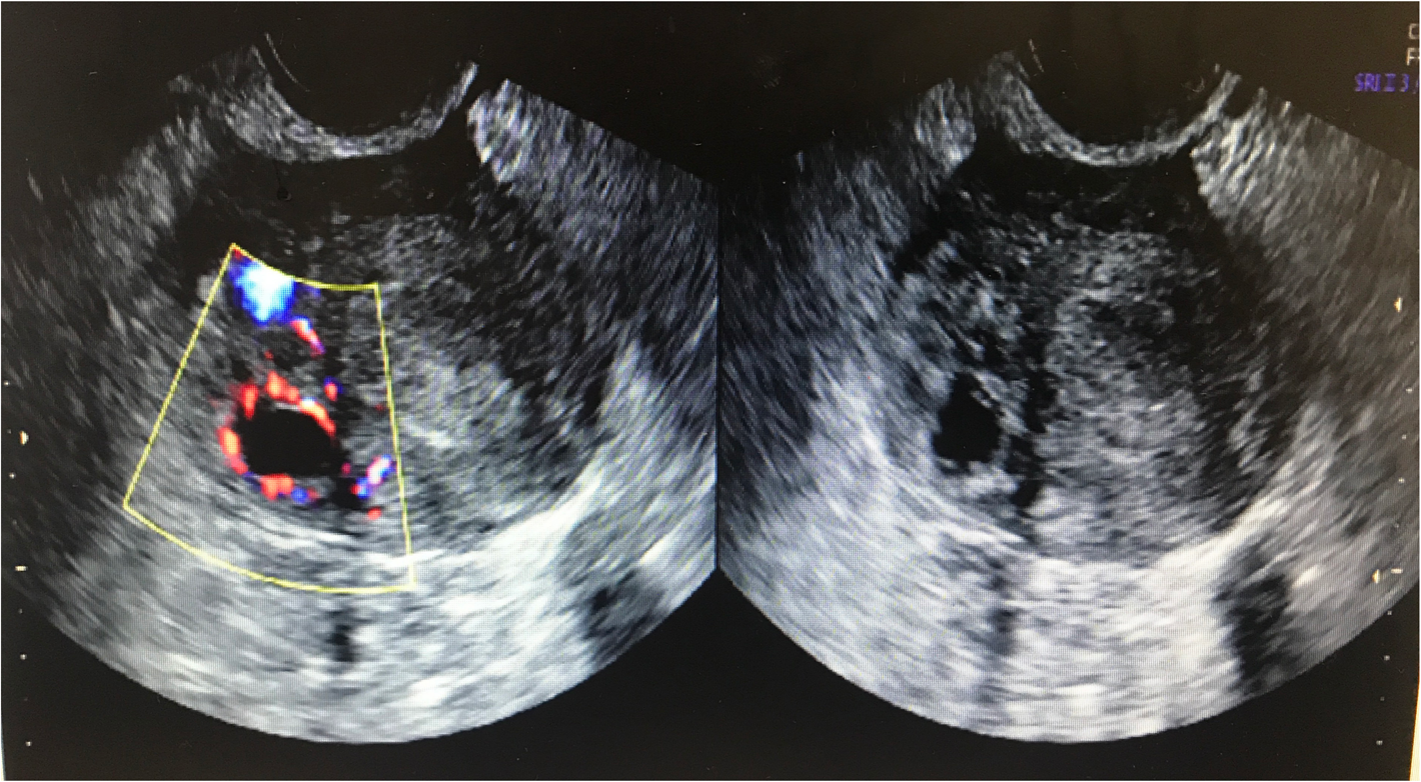
Transvaginal ultrasonography showed the gestation located in the right uterus cornu

Five months later, the patient was undertaken another 2 good quality embryos transfer, but it ended up with chemical pregnancy. 7 months later, the patient was undertaken another 2 embryos transfer. Transvaginal ultrasonography demonstrated a right corneal pregnancy at 6 weeks gestation, suggestive of the recurrence of intramural pregnancy (Fig. 3). The lesion was resected and hysteroplasty surgery was performed under laparoscopy. The serum β-hCG was monitored at intervals until it dropped within normal range.

## Discussion and conclusions

Here we reported an intramural pregnancy after IVF-ET treatment in a patient with a history of right side ipsilateral salpingectomy. We didn’t recognize it as intramyometrial pregnancy from ultrasound because of its untypical manifestation. We proceeded with exploratory laparoscopy and hysteroscopy to clarify the diagnosis, managed intervention in time and preserved fertility at 7 weeks gestation. This case report leaves open for discussion about the etiology, early diagnosis and treatment of intramyometrial pregnancy, especially in women in reproductive age.

ART plays an essential role in the occurrence of ectopic pregnancy [7]. High risk factors associated with ART induced ectopic pregnancy are tubal infertility, multiple embryos transferred, fresh embryo transfer relative to cryopreserved embryo transfer [8], cleavage stage embryo transfer relative to blastocyst stage embryo transfer [9]. Nowadays intramyometrial pregnancy is not as rare as previously believed, as more cases are recognized [6]. However, each patient is in different situation and cases associated with ART published so far are rare [10–12]. The etiological factors of intramyometrial pregnancy include previous uterine trauma, like hysteromyomectomy [11, 13], previous curettage [14–17], cesarean delivery [6], salpingectomy [18], manual removal of placenta, and especially IVF-ET procedure, resulting in a microscopic sinus tract within the endometrium. Embryo may implant into the myometrium with ectopic endometrial tissues during endometriosis development [19–21]. During IVF-embryo transfer, embryo could externally migrate and uterine could perforate due to the active trophoblastic in assisted reproductive treatment [10]. In the case presented, we suspected that the implantation located near the ostium of right fallopian and embedded within the myometrium. The retrograde migration of the embryo through a tract resulting from the right side salpingnectomy previously was one of the possibilities. Another scenario was the implantation on the intramural adenomyosis of the right posterior uterine under the right cornu. There is also possibility reported that embryo transfer catheter penetrated the endometrium or dilated the micro-fistulous tract [22].

Early diagnosis of intramyometrial pregnancy is not easy due to its various manifestation. Diagnosis was sometimes made based on post-operative pathology result [15, 23]. Some of cases were not found until the rupture of the conception [6, 10]. There are a few reports that intramyometrial pregnancy lasts longer than 12 weeks’ gestation [12, 24–27]. With no early recognition and treatment, the gestational sac grows to bulge from the serosal surface of the uterus with progressive thinning of the myometrium. Only two cases in literature have been reported that gestation continued without rupture until over 30 weeks’ gestation with neonatal survival [23, 25]. Delayed diagnosis leads to uterine rupture, hysterectomy, maternal hemoperitoneum, hypovolemic shock, and other life threatening consequences [13].

Ultrasonography is usually the first-time imaging technique to suspect intramyometrial pregnancy diagnosis [5, 28]. Although ultrasound findings might vary according to the location of the gestational sac and the duration of pregnancy, it is possible to diagnose it preoperatively. Some research suggested the ultrasound criteria diagnosis of intramyometrial pregnancy. (1) A gestational sac located above the internal os and medial to the interstitial tube. (2) Trophoblast invasion extends beyond the endometrium-myometrial junction and the conception is partially or completed embedded within the myometrium, which sometimes helps the differential diagnosis with trophoblast disease. Trophoblast disease usually didn’t show a clear boundary between endometrium and gestational trophoblast and it should be considered if β-hCG continues to exceed 10000mIU/l or do not decrease post-partum or post abortion. (3) Lack of decidual reaction in the area of trophoblast, as no obvious white circle sign. (4) Elevated peritrophoblastic blood flow with low resistance [28]. The abundant blood signal around the mass detected and the clinical symptoms that amenorrhea, increasing serum β-hCG and vaginal bleeding are helpful in differential diagnose with uterus fibroid. The risk factors for interstitial pregnancy and corneal pregnancy includes previous ipsilateral salpingectomy, whereby a “tube stump” turns to be the focus of trophoblast implantation [3]. The mobile gestational sac separated from the uterus but surrounded by myometrium and attached to cornu suggested corneal pregnancy, which is one specific kind of intramyometrial pregnancy. Ultrasonography of interstitial pregnancy showed a gestation sac located in the intramyometrial portion of tube surrounded by continues endometrium. Ackerman et al. described “transvaginal ultrasonography interstitial line sign” as a good indicator of interstitial pregnancy, which is an echogenic line between gestational sac surrounded by the thining myometrium mantle and the endometrial cavity [3, 29]. Sometimes it is difficult to differentiate tube interstitial pregnancy from uterus intramural pregnancy especially located in the proximity of the uterine horn. Some authors even use the term corneal pregnancy interchangeably with interstitial pregnancy. In some circumstances it is not certain to diagnose until laparoscopy was performed. In this case, during laparoscopy it was difficult to tell the location of the right tube ostium or if the conception was developed in the intramural portion of the proximal right tubal. So we couldn’t fully exclude interstitial pregnancy during the surgery. But we tended to diagnosis it intramural pregnancy as the conception was located right side of the posterior uterine wall and seemingly not connected to the right fallopian stump nor endometrial cavity. Additionally, from the hysteroscopy we didn’t see the sigh of gestation sac, but the ostiums of both tubes. The intramyometrial pregnancy in our case was not found until laparoscopy in 7 weeks’ gestation.

Clinicians should be aware of different management options and it should be tailored to individual patient. Adaptive treatment option should be selected depending on myometrial involvement, gestational weeks, patient status, and desire for future fertility. Some of reported cases were managed by hysterectomy due to a delayed diagnosis [13, 23]. Conservative management including surgical enucleation and preserved fertility was rare [6, 23, 30]. In our case. Though fertility sparing surgical excision was successfully performed, serious consequences like the ruptured lesion, hypovolemic shock could have happened to this patient. Since the gestation local blood flow is abundant with potential catastrophic hemorrhage, extra caution is needed when regulating bleeding after removing the lesion. Laparoscopic approach with wedge resection requires accurate hemostatic control and minimal tissue trauma. Vasoactive agent injection like potassium chloride into the gestation, suture techniques including purse-string, square, encircling, tourniquet, have all been proposed [31]. Although no strong evidence supports the optimal hemostatic technique because laparoscopy is always an operator-dependent skill, it is essential to balance the adequate bleeding control and avoiding unnecessary tissue dissection and suture placement below myometrial resection which might alter anatomy and hamper fertility [32]. Laparoscopic double-impact devascularization has been reported to be successful management of corneal pregnancy without causing unnecessary tissue dissection and trauma [31]. The main concern with laparoscopic treatment of intramural pregnancy is the subsequent risk of uterine rupture and the recurrence of ectopic pregnancy, which happened to the patient in our case. Transfemoral temporary aortic balloon occlusion, reversible Hem-o-Lok clip occlusion of uterine artery and uterine artery embolization (UAE) have also been reported to prevent extensive bleeding [17, 26, 33–36]. Nevertheless, special attention must be paid to reduce complications such as ischemia, reperfusion injury and thromboembolism [37]. The risk of loss of ovarian reserve in UAE due to the non-target embolization into ovarian arteries has to be considered [38, 39]. Additionally, MTX systemically administered, intramuscular injected or in situ injected, used alone or in combination with laparoscopy, has long been advocated as an alternative method resolving ectopic pregnancy [40–43]. Some research suggested MTX didn’t exert significant adverse effect on fertility in both spontaneous fertile and infertile population [44, 45]. In the case presented, we didn’t choose MTX-based treatment to reduce trophoblast activity at the first place. Early surgical intervention was expected to diagnose and treat the disease simultaneously. We obtained hemostatic control with arginine vasopressin injection and cautiously electrocoagulation though thermal damage to endometrium could be developed.

Though no single universal treatment for intramural pregnancy exists, carefully applied and high resolution transvaginal ultrasonography can help early diagnosis, make fertility sparing treatment possible, and prevent severe complications. It is important for assisted reproductive clinicians to be aware of the risk factors and maintain the index of suspicion of intramural pregnancy, including tubal and cervical ectopic pregnancy, and prevent potentially catastrophic event in its early ages.

## Data Availability

All data generated or analysed during this study are included in this published article.

## Abbreviations

ART: Assisted reproductive technology
CET: Cryopreserved embryo transfer
HRT: Hormone replacement treatment
IVF-ET: In vitro fertilization and embryo transfer
MTX: Methotrexate
OHSS: Ovarian hyper stimulation syndrome
UAE: Uterine artery embolization
